# SG-APSIC1122: Observational study of handwashing sink activities in the inpatient setting

**DOI:** 10.1017/ash.2023.44

**Published:** 2023-03-16

**Authors:** Xiaowei Huan, Sharifah Farhanah, Kyaw Zaw Linn, Clara Chong Hui Ong, Liang Hui Loo, Allie Yin Lim, Nur Hafizah Binte Hamad, Chu Ying Poon, Hui Ru Tan, Ying Wei Tang, Brenda Sze Peng Ang, Marimuthu Kalisvar

**Affiliations:** National Centre for Infectious Diseases, Singapore; Infectious Disease Research and Training Office, National Centre for Infectious Diseases, Singapore; National Public Health & Epidemiology Unit, National Centre for Infectious Diseases, Singapore; Infectious Disease Research and Training Office, National Centre for Infectious Diseases, Singapore; Infectious Disease Research and Training Office, National Centre for Infectious Diseases, Singapore; National Public Health & Epidemiology Unit, National Centre for Infectious Diseases, Singapore; National Public Health & Epidemiology Unit, National Centre for Infectious Diseases, Singapore; National Public Health & Epidemiology Unit, National Centre for Infectious Diseases, Singapore; National Public Health & Epidemiology Unit, National Centre for Infectious Diseases, Singapore; National Public Health & Epidemiology Unit, National Centre for Infectious Diseases, Singapore; Infectious Diseases, Tan Tock Seng Hospital, Singapore; Infectious Diseases, Tan Tock Seng Hospital, Singapore

## Abstract

**Objectives:** The use of handwashing sinks for activities other than hand hygiene (HH) is associated with higher rates of β-lactamase–producing Enterobacteriaceae. However, little has been published about the handwashing sink activities in Singapore hospitals. We explored the handwashing sink activities in a tertiary-care hospital in Singapore. **Methods:** Five trained shadow observers conducted this observational study between December 18 and 21, 2018 (6 hours per day: 07:00–09:00, 09:30–11:30, and 12:30–14:30) in acute-care general wards. We divided the handwashing sink activities by healthcare workers (HCWs) and non-HCWs (ie, visitors, caregivers, and relatives) and by HH- and non–HH-related activities. We used Stata version 15 software for the analysis. The study was approved by the Institutional Review Board of the National Healthcare Group, Singapore (DSRB no. 2020/01257). **Results:** In total, 657 handwashing sink activities were recorded [HCWs, 475 (72.3%) and non-HCWs, 182 (27.7%)]. Of the 475 HCW handwashing sink activities, 451 (94.9%) were HH-related, 10 (2.1%) were for patient nutrition, 7 (1.5%) were for environmental care, 6 (1.3%) were for medical equipment cleaning, and 1 (0.2%) was patient personal-item cleaning. Of the 182 handwashing sink activities by non-HCWs, 117 (64.3%) were HH related, 30 (16.5%) were for patient nutrition, 21 (11.5%) were for personal hygiene, 14 (7.7%) were patient personal-item cleaning. The distribution of handwashing sink activities differed significantly (*P* < .01) between HCWs and non-HCWs. The odds of non–HH-related handwashing sink activities among non-HCWs was 10× higher than among HCWs (OR, 10.44; 95% CI, 5.98–18.23; *P* < .01). **Conclusions:** Handwashing sinks use for non–HH-related activities is higher among non-HCWs than HCWs. Further studies are needed to understand the impact of non-HH handwashing sink activities on nosocomial infections and ways to reduce them.

